# Adjuvant immunotherapy in the modern management of resectable melanoma: current status and outlook to 2028

**DOI:** 10.1016/j.esmoop.2025.104295

**Published:** 2025-02-15

**Authors:** M. Donia, H. Jespersen, M. Jalving, R. Lee, H. Eriksson, C. Hoeller, M. Hernberg, I. Gavrilova, L. Kandolf, G. Liszkay, H. Helgadottir, A. Zhukavets, D. Pianova, I. Marquez-Rodas, B. Neyns, H. Westgeest, I. Pourmir, P. Sobczuk, E. Ellebaek, T. Amaral

**Affiliations:** 1 National Center for Cancer Immune Therapy, Department of Oncology, Copenhagen University Hospital, Herlev, Denmark; 2 Department of Oncology, Oslo University Hospital, Oslo, Norway; 3 University Medical Centre Groningen, Groningen, The Netherlands; 4 Faculty of Biology, Medicine and Health, The University of Manchester, Manchester, UK; 5 Department of Medical Oncology, The Christie NHS Foundation Trust, Manchester, UK; 6 Department of Oncology-Pathology, Karolinska Institutet, Stockholm, Sweden; 7 Theme Cancer Skin Cancer Center, Karolinska University Hospital, Stockholm, Sweden; 8 Department of Dermatology, Medical University of Vienna, Vienna, Austria; 9 Helsinki University Hospital Comprehensive Cancer Center, Helsinki, Finland; 10 Helsinki University, Helsinki, Finland; 11 University Hospital for Active Treatment of Oncology, Sofia, Bulgaria; 12 Faculty of Medicine, Military Medical Academy, Belgrade, Serbia; 13 National Institute of Oncology, Budapest, Hungary; 14 Middle-Eastern European Academy, Budapest, Hungary; 15 Department of Oncology, Belarusian State Medical University, Minsk, Belarus; 16 Riga Stradins University, Riga, Latvia; 17 Medical Oncology Department, Hospital General Universitario Gregorio Marañón, Universidad Complutense, Madrid, Spain; 18 Universitair Ziekenhuis Brussel (UZ Brussel), Department of Medical Oncology, Brussels, Belgium; 19 Amphia Hospital, Department of Internal Medicine, Breda, The Netherlands; 20 European Georges Pompidou Hospital, Department of Thoracic Oncology, Paris, France; 21 INSERM U970, Immunotherapy and Antiangiogenic Treatment in Oncology, Paris, France; 22 Maria Slodowska-Curie National Research Institute of Oncology in Warsaw, Department of Soft Tissue/Bone Sarcoma and Melanoma, Warsaw, Poland; 23 Center for Dermato-oncology, Department of Dermatology, Eberhard Karls University of Tübingen, Tübingen, Germany; 24 Cluster of Excellence iFIT (EXC 2180) “Image-Guided and Functionally Instructed Tumor Therapies”, Tübingen, Germany

**Keywords:** melanoma, adjuvant therapy, immunotherapy, neoadjuvant therapy, approval

## Abstract

**Background:**

Therapeutic advances have reshaped the treatment landscape for patients with resectable melanoma, particularly for those with stage IIB/C and stage III disease. In this article, we discuss the current status and future outlook of adjuvant immunotherapy for melanoma in Europe.

**Results:**

Adjuvant immunotherapy offers significant benefits in terms of recurrence-free survival and distant metastasis-free survival. Uncertainties regarding overall survival (OS) benefits, however, remain. Trials such as Keynote-054, which are expected to provide crucial OS information, have delayed their final analyses until 2027. Additionally, real-world studies have raised questions about the correlation between recurrence-free survival/distant metastasis-free survival improvements observed in clinical trials and OS outcomes in routine clinical practice. These uncertainties have led to ongoing debates about the cost-effectiveness of adjuvant therapies, with disparities in reimbursement policies across Europe reflecting these concerns.

**Conclusion:**

Looking ahead to 2028, adjuvant immunotherapy will remain a key option of comprehensive melanoma care, particularly for patients with stage IIB/C and stage III with micrometastatic disease, where neoadjuvant immunotherapy is not feasible.

## Highlights

• Recent trial results reshaped the standard of care for high-risk melanoma patients.
• Neoadjuvant ± adjuvant treatment is currently available for clinically detected resectable stage III.
• The role of adjuvant anti-PD-1 therapy for patients with micrometastatic stage III disease requires continuous re-evaluation.

## Introduction

The field of resectable melanoma treatment has witnessed recent transformative advances, particularly based on the results of neoadjuvant anti-programmed cell death protein 1 (PD-1)-based immunotherapy trials for resectable macroscopic stage III or IV melanoma.^1^^,^^2^ These advances are reshaping standard-of-care protocols for a proportion of patients with high-risk melanoma. Amidst these promising developments, however, it is essential to continuously re-evaluate the role of adjuvant anti-PD-1-based immunotherapy in managing patients with stage IIB/C or stage III with micrometastatic disease.

## Neoadjuvant immunotherapy versus adjuvant immunotherapy in stage ≥IIIB melanoma: Two indications for two distinct patient populations

Clinically occult nodal disease in melanoma is characterized by the presence of cancer cells in the lymph nodes that are not detectable on clinical or radiological examination, but can be identified through microscopic examination following sentinel node biopsy.^3^ This stage of the disease escapes the eligibility criteria for neoadjuvant therapy trials,^1^^,^^2^ which generally select patients with macroscopic nodal disease with lesions that are palpable, >1-1.5 cm or detectable by diagnostic imaging such as ultrasound, computed tomography or positron-emission tomography scans, and can be biopsied. Consequently, patients with clinically occult disease are currently candidates for adjuvant immunotherapy post-surgery, and not neoadjuvant immunotherapy pre-surgery. In this patient population, the benefit of adjuvant anti-PD-1 immunotherapy to reduce the risk of recurrence and the development of distant metastases was established in multiple phase III trials where either nivolumab or pembrolizumab were tested against observation^4^, ^5^, ^6^ or against the pre-anti-PD-1 standard of care immunotherapy (ipilimumab or interferon alpha).^7^^,^^8^

While it is clear that a proportion of patients with stage ≥IIIB melanoma are not eligible to current neoadjuvant immunotherapy, the size of this patient population has not been established so far. Data from the Danish Metastatic Melanoma Database (DAMMED), a nationwide database collecting information on all patients evaluated for systemic adjuvant treatment in Denmark,^9^ can provide an initial estimation. Here, of all patients with resected stage ≥IIIB melanoma evaluated for systemic adjuvant treatment, 40.6% were diagnosed with TNM (tumor–node–metastasis) classifications indicating clinically occult nodal disease (N1a, N2a, N3a) and no distant metastasis (M0) (source Eva Ellebaek, DAMMED). These data demonstrate that a substantial subset of patients, not eligible for neoadjuvant immunotherapy but who may benefit from adjuvant treatment, remains. This figure, however, is likely to be significantly higher than 40% in practice, due to a number of factors affecting the definition of macroscopic versus microscopic disease. For example, a proportion of patients with non-N1a, N2a, or N3a disease may present with non-macroscopic microsatellite metastases (N1c, N2c, or N3c), with radiologically detected suspect lesions (N1b, N2b/c, or N3b/c) that are not amenable to pre-immunotherapy histological confirmation, or with lesions that are not sufficiently large to meet local requirements for initiation of neoadjuvant or perioperative immunotherapy. This information is not captured by the current American Joint Committee on Cancer (AJCC) staging, preventing a precise estimation of the proportion of patients with stage ≥IIIB melanoma who are not eligible for neoadjuvant treatment.

Overall, in the immediate future adjuvant treatment is expected to maintain its cornerstone role for a sizable proportion (>40%) of patients with stage ≥IIIB melanoma, in addition to the form indication in stage IIB/C (only primary tumor) and IIIA (micrometastatic regional nodal disease). In addition, while the need for adjuvant immunotherapy (pathological response driven as in NADINA, or for all as in SWOG S1801) following neoadjuvant immunotherapy is still unclear, it is likely that adjuvant immunotherapy will also retain an add-on role for some patients after neoadjuvant treatment.

The future of adjuvant immunotherapy post-2028 is more uncertain, however, as studies testing neoadjuvant immunotherapy in earlier stages of disease, such as intradermal anti-PD-1 plus anti-cytotoxic T lymphocyte-associated antigen-4 (CTLA-4) (https://www.clinicaltrials.gov/study/NCT06240143) or systemic anti-PD-1 plus anti-LAG3 (https://clinicaltrials.gov/study/NCT05418972) in high-risk stage II melanoma, are undergoing.

## Status of adjuvant immunotherapy for melanoma

The approval of nivolumab and pembrolizumab for resected melanoma in 2018-2019 by the European Medicines Agency (EMA) marked a transformative change in melanoma treatment. These approvals extended to patients with stage III and IV (only nivolumab) resected melanoma. More recently in 2022-2023, the same drugs were approved by EMA for resected stage IIB/C melanoma, further broadening the therapeutic scope of adjuvant immunotherapy in selected countries. Therefore, across most European countries, evaluation of patients for adjuvant anti-PD-1-based immunotherapy after complete melanoma resection is a cornerstone of the shared decision-making strategy for patients with stage ≥IIB melanoma, i.e. for all patients with stage IIB/C and IIIA melanoma, and patients with non-macroscopic stage ≥IIIB melanoma.

The rationale for offering anti-PD-1-based adjuvant immunotherapy is supported by data demonstrating significant enhancements in recurrence-free survival (RFS) and distant metastasis-free survival (DMFS), with consistent hazard ratios (HR) for RFS in favor of anti-PD-1 immunotherapy versus placebo at ∼0.6, meaning ∼40% relative reduction of the risk of a composite event defined by recurrence or death.^4^, ^5^, ^6^, ^7^ Significant uncertainties remain due to the lack of direct evidence from phase III clinical trials for improved overall survival (OS) for adjuvant immunotherapy at any stage, however, possibly introduced by the prospect of long-term benefit of anti-PD-1 based therapy upon unresectable relapse. Notably, the final OS results for Keynote-054, a trial specifically designed to answer this question for patients with resected stage III melanoma and initially expected in 2023 (based on the initial post-authorization efficacy study plan approved by EMA^10^ and postmarketing commitment study plan approved by the Food and Drug Administration^11^), have been delayed until Q4-2027 according to the most recent EMA summary of product characteristics (SmPC) for pembrolizumab.^12^ Additionally, the Checkmate-238 trial up to its seventh year of follow-up, showed no statistically significant OS benefit for nivolumab versus ipilimumab, despite nivolumab demonstrating improved RFS and DMFS,^7^ highlighting the possibility of a weak or lack of association between RFS/DMFS benefits and OS benefits, especially when patients have access to active treatment options including anti-PD-1-based therapies at the time of unresectable recurrence. Similar results were observed in SWOG S1404, where patients with resected, high-risk stage III and stage IV melanoma were randomized to pembrolizumab or standard-of-care (at the time of trial conduct) high-dose interferon-α2b or ipilimumab, with statistically significant improvement in RFS but not OS.^8^ Furthermore, no OS benefit of adjuvant nivolumab was observed in the IMMUNED trial.^13^ In all these trials OS was a secondary endpoint, except SWOG S1404 where OS was a co-primary endpoint. A Swedish national real-world retrospective study also reported a lack of OS benefit for patients diagnosed after (July 2018-December 2020) versus before (January 2016-June 2018) the introduction of adjuvant therapies in Sweden with >3 years median follow-up and 64% of included patients in the post-adjuvant cohort who received adjuvant therapies.^14^^,^^15^ In contrast, indirect analyses using data from two different trials, such as the one involving nivolumab versus ipilimumab and ipilimumab versus placebo, have suggested an OS benefit.^16^ A difference, however, in availability to anti-PD-1-based therapies at the time of recurrence between these two trials that were conducted at different time points needs to be taken into consideration. Additionally, a real-world retrospective study using the United States National Cancer Database (NCDB) indicated a survival advantage for patients diagnosed in 2016-2020 with stage IIIB, IIIC, and IIID who received postoperative immunotherapy, but not for patients with stage IIIA.^17^ These studies provide, by their design, however, weaker levels of evidence than registrational randomized controlled phase III trials and might thus be deemed insufficient to confirm OS benefit by health technology assessment (HTA) agencies.

## Adjuvant immunotherapy for melanoma in europe

**Figure 1:**
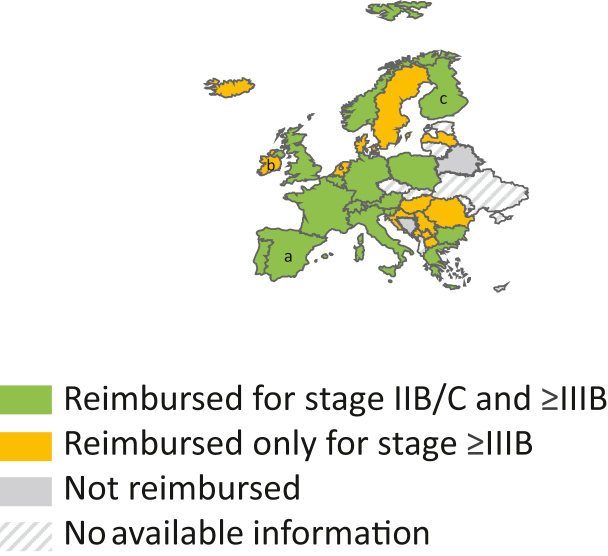
Reimbursement of adjuvant anti-PD-1 for melanoma across Europe. Information to generate Figure 1 was provided by the authors of this article or by internet search in the official websites of national healthcare agencies in August 2024. Figure template was obtained from https://slidelizard.com/en/blog/powerpoint-world-map (accessed on 1 July 2024). Geographical borders of Europe were drawn by the corresponding authors. PD-1, programmed cell death protein 1. ^a^Not reimbursed for stage IIIB. ^b^Full pharmacoeconomic assessment for stage IIB/C ongoing. ^c^Reimbursed for stage IIC but not IIB.

The current status on the availability of adjuvant anti-PD-1 immunotherapy in most European countries, based on national reimbursement policies, is shown in Figure 1.

While reimbursement for resected stage ≥IIIB is broadly available in most European countries following positive evaluations from several HTA agencies, the status for reimbursement of disease sub-staging (e.g. stage IIIA) or stage IIB/C is more heterogeneous.

The lack of direct evidence from phase III trials for OS benefit leading to uncertain correlation between RFS and OS until (presumably) 2028, combined with discrepancies in OS improvements from retrospective studies in stage III melanoma, may have three important sets of consequences:

• Reimbursement of adjuvant anti-PD-1 immunotherapy for stage IIB/C and stage III melanoma: despite similar percentage risk reductions for RFS and DMFS as with stage III, in phase III clinical trials patients with resected stage IIB/C had an overall lower absolute risk of recurrence (with IIC having a worse outcome in terms of RFS than IIB).^5^^,^^18^ This leads to a potentially higher number of patients needed to treat to prevent a recurrence, while at the same time exposing these patients to potential toxicity.^19^ Of note, it was estimated that five to nine patients with stage IIB and four to seven patients with stage IIC would need to be treated to prevent one recurrence (https://link.springer.com/article/10.1245/s10434-024-16418-y), with additional uncertainties regarding the case of patients with stage IIIA and low (<1 mm) sentinel node tumor burden, especially for those with very low (<0.3 mm) tumor deposits (https://pubmed.ncbi.nlm.nih.gov/35849790/).
In addition, evaluation by HTA agencies for reimbursement of anti-PD-1 for stage IIB/C melanoma may be additionally influenced by the lack of direct evidence for OS improvements in stage III melanoma coupled with uncertain association between positive RFS and OS, leading to uncertain societal benefits. While each HTA agency may have different requirements, OS is considered a critical endpoint in trials of adjuvant treatment^20^ and often used as a benchmark in decision for reimbursement of oncology drugs. As a notable example, the Danish Medicines Council specifically did not recommend anti-PD-1 (pembrolizumab) for stage IIB/C melanoma given the lack of known OS benefit and lack of documented positive correlation between RFS and OS,^21^ and connected the reimbursement of pembrolizumab for resected stage III melanoma to a re-evaluation in 2027,^22^ with a similar re-evaluation to be conducted in the Netherlands^23^ and Sweden.^24^ Therefore, the reimbursement of adjuvant immunotherapy for melanoma, particularly stage IIB/C, may be subject to additional considerations on cost-effectiveness until 2028.
• Utilization of adjuvant anti-PD-1 immunotherapy for stage IIB/C and stage III melanoma: Current clinical practice guidelines and consensus guidelines of the European Society of Medical Oncology (updated 2018 and 2020) endorse treatment of most patients with resected stage III melanoma,^25^ and immunotherapy for stage IIB/C melanoma is endorsed by the American Society for Clinical Oncology guidelines (updated 2023).^26^ Uncertainties related to OS, however, may have significant consequences in anti-PD-1 immunotherapy utilization over the next years. The inability to provide comprehensive information to patients on the impact on survival of an offered treatment (coming with potentially life-threatening and long-term toxicities) may lead to gaps in the informed, shared decision-making between patient and clinician. The absence of a clear evaluation of the pros and cons of adjuvant immunotherapy in the long run may lead to a reduction in the utilization of adjuvant immunotherapy in the coming years due to the uncertainty about whether it improves OS, perhaps the most critical outcome together with quality of life that ‘matters’ for patients diagnosed with cancer.^27^ In fact, trends in the reduction of adjuvant immunotherapy usage—especially for patients with lower-risk disease—can already be observed in 2023 in one European country (Table 1). Notably, from the perspective of patient preference, prolonging the time to (or preventing) recurrence or to developing distant metastasis—even in the absence of direct evidence for OS benefit—may also be considered a valuable outcome. Of note, in a real-world study, >50% of first recurrences for patients with stage ≥IIB melanoma were located at distant metastatic sites.^28^ Given the speed of drug development, delay in recurrence may also lead to the availability of more effective treatments at time of unresectable disease recurrence.^28^ Therefore, where available, adjuvant anti-PD-1 immunotherapy is expected to remain a fundamental component of the shared decision-making process for most patients.

**Table 1:** Proportion of all patients assessed who received adjuvant therapy among residents in three Danish regions^a^

Disease stage	Year of diagnosis				
	2019	2020	2021	2022	2023
IIIA	41%	43%	37%	41%	18%
IIIB	73%	75%	74%	78%	62%
IIIC	80%	72%	71%	72%	68%

a DAMMED contains population-based information of all patients to be evaluated for systemic adjuvant therapy for only three out of five Danish regions, covering 67% of the Danish resident population. Under 5% of patients treated with adjuvant therapy have received adjuvant dabrafenib plus trametinib (source: DAMMED; annual report 2023).
• A potential shift towards adjuvant targeted therapy in resected stage III *BRAF* V600E mutant melanoma: in a phase III trial, adjuvant therapy with dabrafenib plus trametinib for patients with resected stage III melanoma has shown significant improvements compared with placebo for RFS, the primary endpoint of the trial, and DMFS (RFS HR 0.52; 95% CI 0.43-0.63, DMFS HR 0.56; 95% CI 0.44-0.71); OS rates were 86% (95% CI 82% to 89%) versus 77% (73% to 82%) at 3 years, 79% (75% to 83%) versus 70% (73% to 82%) at 5 years, 73% (68% to 77%) versus 66% (62% to 71%) at 7 years, although no statistically significant OS benefit was demonstrated (HR 0.80; 95% CI 0.62-1.01; *P* = 0.06).^29^ Paradoxically, a smaller subgroup analysis of patients with a BRAF V600K mutant melanoma treated with dabrafenib plus trametinib suggested that this subgroup may obtain inferior survival compared with placebo, while RFS was improved. There has been no head-to-head trial of targeted therapy compared with anti-PD-1 immunotherapy in the adjuvant setting. Data from real-world studies would suggest that dabrafenib plus trametinib have similar or better efficacy in terms of RFS and DMFS, although these have short follow-up and come with the caveats of retrospective analyses.^30^, ^31^, ^32^, ^33^ Therefore, in the shared decision-making process for adjuvant immunotherapy versus targeted therapy, the toxicity profile of these strategies has a pivotal role. Due to the decreased risk of chronic toxicity, particularly endocrinopathies, targeted therapy may be potentially preferred by some patients and clinicians. Real-world, post-authorization studies will be important to describe a potential treatment pattern evolution.

Future scenarios, likely after 2028, should take into consideration several additional unknown variables, primarily related to the results of ongoing studies of adjuvant treatment such as those testing anti-PD-1 plus anti-LAG3 (https://clinicaltrials.gov/study/NCT05608291) or anti-PD-1 plus a personalized neoantigen vaccine (https://clinicaltrials.gov/study/NCT05933577).

## Conclusions

Maturation of clinical trial data complemented by real-world studies will undoubtedly refine our understanding of the utility of adjuvant anti-PD-1 immunotherapy treatment approaches. Given the major OS benefits provided by immune checkpoint inhibitors in metastatic melanoma, particular attention to post-recurrence treatments in the control arms of randomized clinical trials is warranted. Current data underscore the necessity of maintaining a dual strategy that includes offering (where available) primarily neoadjuvant therapy for patients with resectable stage III macroscopic disease, and adjuvant therapies to patients with resected stage ≥IIB melanoma. A careful shared decision-making process with the individual patient is essential to assess the pros and cons of starting adjuvant anti-PD-1-based immunotherapies given the current lack of clear OS benefit, both for adjuvant as well as neoadjuvant treatment regimens. While uncertainties surrounding OS outcomes persist and may impact the utilization of adjuvant anti-PD-1 across Europe until 2028, especially for patients with a lower risk of recurrence, clinicians can integrate meaningful endpoints such as RFS and DMFS into the decision-making framework. These surrogate endpoints provide a valuable basis for guiding treatment discussions, particularly for patients with high-risk disease.

In conclusion, the case for adjuvant immunotherapy as a means to improve RFS and DMFS remains robust, especially for the thousands of patients with stage IIB/C and stage III melanoma who present with micrometastatic disease and value these endpoints. As such, it currently remains an important option to discuss in the shared decision-making process of comprehensive melanoma care, ensuring that all patients receive the most judicious and effective treatment options based on their specific disease characteristics and personal preferences.
