# Corrigendum to “Adjuvant immunotherapy in the modern management of resectable melanoma: current status and outlook to 2028” [ESMO Open 10 (2025) 104295]

**DOI:** 10.1016/j.esmoop.2025.105492

**Published:** 2026-01-02

**Authors:** M. Donia, H. Jespersen, M. Jalving, R. Lee, H. Eriksson, C. Hoeller, M. Hernberg, I. Gavrilova, L. Kandolf, G. Liszkay, H. Helgadottir, A. Zhukavets, D. Pjanova, I. Marquez-Rodas, B. Neyns, H. Westgeest, I. Pourmir, P. Sobczuk, E. Ellebaek, T. Amaral

**Affiliations:** 1 National Center for Cancer Immune Therapy, Department of Oncology, Copenhagen University Hospital, Herlev, Denmark; 2 Department of Oncology, Oslo University Hospital, Oslo, Norway; 3 University Medical Centre Groningen, Groningen, The Netherlands; 4 Faculty of Biology, Medicine and Health, The University of Manchester, Manchester, UK; 5 Department of Medical Oncology, The Christie NHS Foundation Trust, Manchester, UK; 6 Department of Oncology-Pathology, Karolinska Institutet, Stockholm, Sweden; 7 Theme Cancer Skin Cancer Center, Karolinska University Hospital, Stockholm, Sweden; 8 Department of Dermatology, Medical University of Vienna, Vienna, Austria; 9 Helsinki University Hospital Comprehensive Cancer Center, Helsinki, Finland; 10 Helsinki University, Helsinki, Finland; 11 University Hospital for Active Treatment of Oncology, Sofia, Bulgaria; 12 Faculty of Medicine, Military Medical Academy, Belgrade, Serbia; 13 National Institute of Oncology, Budapest, Hungary; 14 Middle-Eastern European Academy, Budapest, Hungary; 15 Department of Oncology, Belarusian State Medical University, Minsk, Belarus; 16 Riga Stradins University, Riga, Latvia; 17 Medical Oncology Department, Hospital General Universitario Gregorio Marañón, Universidad Complutense, Madrid, Spain; 18 Universitair Ziekenhuis Brussel (UZ Brussel), Department of Medical Oncology, Brussels, Belgium; 19 Amphia Hospital, Department of Internal Medicine, Breda, The Netherlands; 20 European Georges Pompidou Hospital, Department of Thoracic Oncology, Paris, France; 21 INSERM U970, Immunotherapy and Antiangiogenic Treatment in Oncology, Paris, France; 22 Maria Slodowska-Curie National Research Institute of Oncology in Warsaw, Department of Soft Tissue/Bone Sarcoma and Melanoma, Warsaw, Poland; 23 Center for Dermato-oncology, Department of Dermatology, Eberhard Karls University of Tübingen, Tübingen, Germany; 24 Cluster of Excellence iFIT (EXC 2180) “Image-Guided and Functionally InstructedTumor Therapies”, Tübingen, Germany

The authors regret that in the original publication the name of Dr Pjanova was spelled incorrectly; the correct spelling is now given above.

Further, for the sentence “Overall, in the immediate future adjuvant treatment is expected to maintain its cornerstone role for a sizable proportion (>40%) of patients with stage ≥IIIB melanoma, in addition to the form indication in stage IIB/C (only primary tumor) and IIIA (micrometastatic regional nodal disease).” please read:

“Overall, in the immediate future adjuvant treatment is expected to maintain its cornerstone role for a sizable proportion (>40%) of patients with stage ≥IIIB melanoma, in addition to the formal indication in stage IIB/C (only primary tumor) and IIIA (micrometastatic regional nodal disease).”

The authors would like to apologise for any inconvenience caused.

